# Predictors of weaning failure in case of VA ECMO implantation

**DOI:** 10.1038/s41598-022-18105-y

**Published:** 2022-08-16

**Authors:** Axelle Cusanno, Nadia Aissaoui, Vincent Minville, Jean Porterie, Caroline Biendel, Kim Volle, Laure Crognier, Jean-Marie Conil, Clément Delmas

**Affiliations:** 1 Department of Anesthesia, Intensive Care and Perioperative Care Medicine, Rangueil University Hospital, Toulouse, France; 2 Medical Intensive Care Unit, Hôpital Cochin, AP-HP, Paris, France; 3 Department of Cardiovascular Surgery, Rangueil University Hospital, Toulouse, France; 4 Intensive Cardiac Care Unit, Department of Cardiology, Rangueil University Hospital, Toulouse, France; 5 Intensive Cardiac Care Unit, Department of Cardiology, Toulouse University Hospital, 1, avenue Jean Poulhès, TSA 50032, 31059 Toulouse Cedex 9, France

**Keywords:** Cardiac device therapy, Interventional cardiology

## Abstract

The use of veno-arterial extracorporeal membrane oxygenation (VA ECMO) for the treatment of refractory cardiogenic shock has increased significantly. Nevertheless, early weaning may be advisable to reduce the potential for severe complications. Only a few studies focusing on ECMO weaning predictors are currently available. Our objective was to evaluate factors that may help predict failure during VA ECMO weaning. We included 57 patients on VA ECMO support previously considered suitable for weaning based on specific criteria. Clinical, haemato-chemical and echocardiographic assessment was considered before and after a “weaning test” (ECMO flow < 2 L/min for at least 60 min). ECMO removal was left to the discretion of the medical team blinded to the results. Weaning failure was defined as a patient who died or required a new VA ECMO, heart transplant or LVAD 30 days after ECMO removal. Thirty-six patients (63.2%) were successfully weaned off VA ECMO, of whom 31 (54.4%) after the first weaning test. In case of first test failure, 3 out of 7 patients could be weaned after a 2nd test and 3 out of 4 patients after a 3rd test. Pre-existing ischemic heart disease (OR 9.6 [1.1–83]), pre-test left ventricular ejection fraction (LVEF) ≤ 25% and/or post-test LVEF ≤ 40% (OR 11 [0.98–115]), post-test systolic blood pressure ≤ 120 mmHg (OR 33 [3–385]), or length of ECMO support > 7 days (OR 24 [2–269]) were predictors of weaning failure. The VA ECMO weaning test failed in less than 40% of patients considered suitable for weaning. Clinical and echocardiographic criteria, which are easily accessible by a non-expert intensivist, may help increase the probability of successful weaning.

## Introduction

Veno-arterial extracorporeal membrane oxygenation (VA ECMO) has become the standard approach for severe hemodynamic following refractory cardiogenic shock (CS) or cardiac arrest (CA) whatever their causes^1,2^. Although ECMO has been a matter of debate given its low level of evidence (IIbC) according to the European and American guidelines), this approach remains appropriate when required and ongoing randomised study may shed some more lights on its role^3,4^ [EURO-SHOCK (NCT03813134), ANCHOR (NCT04184635), and ECLS-SHOCK (NCT03637205)].

Although improved ECMO technology has enabled widespread use to non-transplant centres, prolongation of support remains associated with significant complications in up to 30–50% of cases such as haemolysis, limb ischaemia, acute pulmonary oedema, haemorrhagic and thrombotic events, sepsis and renal failure^1,2,5,6^.

Therefore, early ECMO weaning may be appropriate to optimize outcome in terms of recovery or bridge to transplantation or long-term left ventricular assist device (LVAD). Nevertheless, early weaning may lead to the need of emergency re-implantation.

Despite a consensus on the practical weaning approach based on gradual flow reduction, timing and weaning criteria have not been completely reviewed to ascertain its effectiveness^7^. Echocardiographic assessment has a major role to play in clinical decision-making, either as a standalone investigation or in association with haemodynamic tolerance criteria^5,6,8–18^. The microcirculation^19^, biological markers of myocardial recovery such as cardiac enzymes and lactate^20–22^ or general prognostic criteria for survival^15,22,23^ have been proposed during ECMO weaning, which remains a challenging approach considering that 20–65% of patients do not reach survival to discharge due to the onset of multi organ failure.

Our objective was to evaluate factors that may help predict failure during VA ECMO weaning. Primary endpoint was weaning failure defined as a patient who died or required new VA ECMO, heart transplant or LVAD 30 days after ECMO removal.

## Materials and methods

### Patients

This observational, prospective, monocentric study was conducted from May 2016 to March 2021, in our tertiary hospital intensive care unit (ICU) (Rangueil University Hospital, Toulouse, France). The study was conducted in accordance with the principles outlined in the Declaration of Helsinki. Patients and/or relatives were informed but the requirement for informed consent was waived by Toulouse University Hospital Research Ethics Committee (no. 94-1214) which approved our protocol study as a non interventional study.

Patients were included if (1) they were under VA ECMO support for refractory CS or CA, and if (2) a VA ECMO weaning test was proposed since they were considered suitable for weaning. Patients were excluded in case of therapeutic limitation measures, brain death or if weaning from ECMO was not an option (patient having end-stage heart failure and waiting heart transplant or LVAD).

### Data collected

At the time of VA ECMO implantation, patient characteristics (age, gender, history), indications for ECMO, position of the VA ECMO cannulae, biological assessment (arterial blood gases and lactates, creatinine, prothrombin time (PT), blood and platelet count) were collected.VA ECMO complications (acute pulmonary edema, limb ischemia, intracerebral hemorrhage, hemorrhagic shock, hemolysis, tamponade,…) as possible left ventricular unloading techniques used (atrioseptostomy, left ventricular unloading cannula, intra-aortic balloon pump (IABP) or Impella) were also noted.

The minimum left ventricular ejection fraction (LVEF) was recorded.

At the time of ECMO weaning, data concerning the length of ICU stay, the duration of mechanical ventilation, and the total duration of inotropes and vasopressors therapies were collected. The lengths of time from ECMO implantation to weaning test and from weaning test to ECMO weaning were also recorded.

Thirty days after weaning, the total length of ICU and in hospitalization stays, total mechanical ventilation time, total vasopressor and inotrope times were collected. Finally, death and its cause, the need of a new VA ECMO support, LVAD or a heart transplant were recorded at 30 days, based on the patient's computerized record or by telephone interview with the attending physician or referring specialists of the patient.

The data used for this analysis and the manuscript are available on request from the corresponding authors.

### Weaning test

Weaning test was performed in patients deemed suitable for weaning according to our local protocol i.e. in case of (1) hemodynamic stability (mean blood pressure (MBP) ≥ 65 mmHg) with blood pressure pulsatility, low doses of catecholamines (noradrenaline < 2 mg/h and dobutamine ≤ 5 μg/kg/min) and (2) respiratory stability (PaO_2_ > 60 mmHg with no clinical or radiological signs of acute respiratory distress syndrome).

The ECMO flow was decreased to 1 to 2 l/min for a period of at least 60 min.

Previous patient course in ICU, usual clinical parameters, ongoing treatments and organ support were recorded before and at the end of the weaning test. A transthoracic echocardiogram (TTE), performed by a cardiologist, recorded parameters of systolic and diastolic left ventricular function, systolic right ventricular function, left and right ventricular pressure before and at the end of the weaning test. Details of clinical and echocardiographic parameters recorded are available in Supplementary data.

The ECMO flow rate was then reset to the initial value and the final decision to wean the patient was left to the discretion of the medical team who were blinded to the weaning test results.

### Statistical analyses

The study population was divided into successful (SWG) or failed (FWG) ECMO weaning groups at 30 days. The statistical analysis after verification of the distribution of the parameters (Shapiro–Wilk test) involved several steps. First of all, descriptive statistics were used to analyze the continuous (medians, [interquartile ranges]) and nominal variables on the entire study population and in each of the two groups. A comparison was then made between the 2 groups of continuous variables, most often by a non-parametric test (Mann–Whitney), and of nominal variables by a Chi-squared test or a Fischer’s exact test.

The clinical, biological, and ultrasonographic parameters were measured for each test during 2 periods, and for each test, the changes in these parameters were examined using a Wilcoxon test. For the qualitative variables, the change during the 2 test periods was evaluated using Cochran's *Q* test.

Therefore, for one test and in particular the first weaning test performed (SEV1), the analysis of each quantitative variable involved 4 comparisons and quantified 4 p: p^1^ = comparison of the parameter of each group: CS (clinical success) vs CF (clinical failure) (Mann–Whitney); p^2^ p^3^ p^4^ = change in the parameter in the 2 test periods for the entire sample and in each group (Wilcoxon). The objective of this approach was the static analysis and comparison of each variable (answer to the question: is the starting level identical during the 2 phases of the test?) as well as the dynamic analysis (is there change in the variable between the first and second phase of the test?).

The discriminative value of each variable of interest in relation to the primary endpoint was assessed by studying ROC curves, prioritizing those with an AUC > 0.8 or including this value in their 95% CI. The most discriminating thresholds were chosen according to the best Youden index. The ROC curves were then compared in order to determine the most discriminating of these variables.

In the final step of the multivariate analysis, the statistically significant parameters (p < 0.05) and those with a p < 0.2 were tested first in a logistic regression model, taking into account the limited number of patients concerned by the event studied (weaning failure). Among the models tested, the one for which the observed data were the best fit was chosen according to 3 criteria: the Hosmer and Lemeshow goodness-for-fit test, the prediction percentage and the ROC curve of the model.

To better identify individuals in the study population sample for whom weaning failed, the patients were clustered by the use of a segmentation tree. The purpose of this technique was to describe the means of distributing the population into homogeneous groups according to the occurrence of weaning failure and previously selected variables for the multidimensional analysis. We tested 2 statistical algorithms for building decision trees, the “CHAID” (Chi-squared Automatic Interaction Detection) and CRT (Classification and Regression Trees) methods, and chose the one for which the forecast percentages were highest. The model was validated by cluster sampling: training sample of 50% of the patients and then a test sample of the remaining patients. The variables validated in these 2 steps were then included and tested on all 57 patients in the overall sample.

The study was conducted on IBM SPSS Statistics Version 23 software (Chicago, IL) for the majority of analyses (http://www.ibm.com/software/analytics/spss/products/statistics/index.html). *p* < 0.05 was considered to be statistically significant.

### Ethics approval and consent to participate

The study was conducted in accordance with the principles outlined in the Declaration of Helsinki. The study was conducted in accordance with the principles outlined in the Declaration of Helsinki. Patients and/or relatives were informed but the requirement for informed consent was waived by Toulouse University Hospital Research Ethics Committee (no. 94-1214) which approved our protocol study as a non interventional study.

## Results

### Characteristics of the study population

Fifty-seven patients who underwent one or more VA ECMO weaning tests were included. Supplementary Table 1 describes the baseline characteristics of the study population. They were predominantly male (n = 37; 65%), with a median age of 50 [38–58] years. A known cardiomyopathy was frequent (n = 37; 65%). Cardiovascular risk factors associated hypertension (n = 14, 25%), active smokers (n = 24, 42%), obesity (n = 9, 16%), dyslipidemia (n = 8, 14%) and diabetes (n = 8, 14%). The main indications for ECMO were ischemic CS (n = 20, 35%) and refractory CA (n = 16, 28%). VA ECMO was mainly implanted through a femoro-femoral approach (83%). The ICU stay was 15 [12–25] days with 11 [8–17] days under mechanical ventilation, 4 [2–7] days under inotropic and 4 [2–7] days under vasopressor supports.

### 30 days outcome

**Figure 1:**
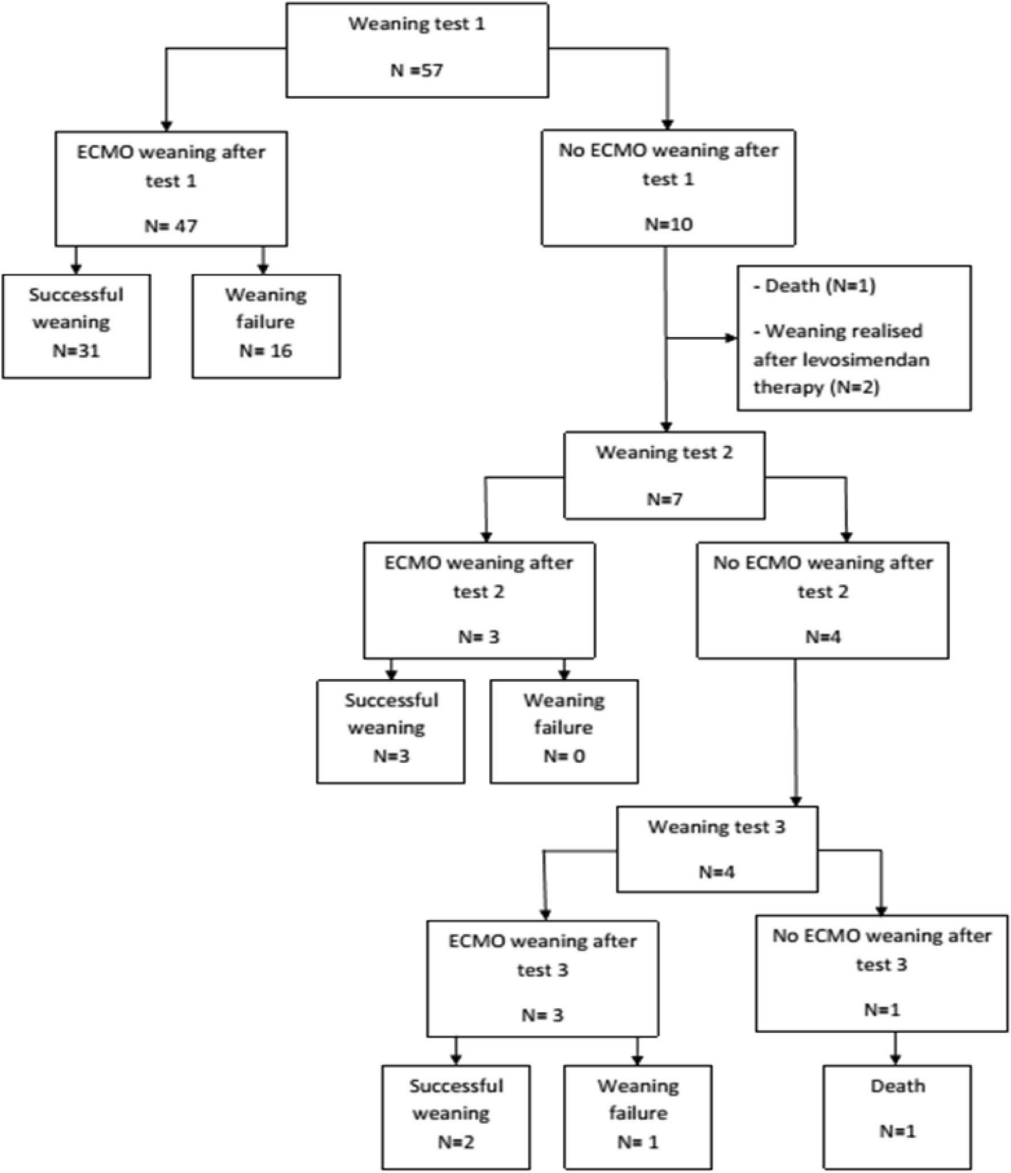
Flow chart of the population. Weaning failure was defined as a patient who died or required a new VA-ECMO, heart transplant or LVAD 30 days after withdrawal from ECMO.

At 30-day, 36 patients (63%) were alive with no need for heart transplantation, LVAD, or a new VA ECMO defining the SWG. Failures were distributed as following: 17 (30%) deaths, 2 (4%) heart transplants, 1 (2%) new VA ECMO and no LVAD placement (Fig. 1). Only one weaning test was performed in the majority of cases (n = 50; 87.7%), but two tests was performed for 7 (12%) and three tests for 4 (7%) patients (Fig. 1). When weaning was carried out it was a success for 31 on 47 (86%) patients after the first test, for 3 patients on 3 (100%) after the second test and for 2 patients on 3 (67%) after the third test (Fig. 1). Given the low number of patients who required multiple tests, their descriptive analyses were not provided.

### Comparative analysis of patient characteristics based on success or failure of the first weaning test

On admission, the two groups (SWG and FWG) were comparable in terms of morphological data, history, indications for VA-ECMO, clinical and biological presentations. In contrast, pre-existing ischemic heart disease was significantly more common in FWG (38 vs 14%; p = 0.037) (Supplementary Table 1). Patients in the FWG had longer median total times on vasopressors (2 [1–4] vs 6 [4–9]; p = 0.006) and under mechanical ventilation (10 [7–16] vs 15 [10–18]; p = 0.033). They underwent more renal replacement therapies (RRT) (3 (4%) versus 8 (38%); p = 0.012), had a tendency for longer ECMO implant-weaning test time, but no significant difference regarding the use of IABP or levosimendan at the time of the weaning test (Table 1 and Supplementary Table 1).

**Table 1:** Comparison of the characteristics of the two groups (successful and failure weaning groups) at the time of weaning test.

	Overall population n = 57			Successful weaning group n = 36		Failure weaning group n = 21				p
	N	Median	25–75 P	N	Median	25–75 P	N	Median	25–75 P	
Implantation-weaning test 1 delay (days)	46	5	4–8	36	4.5	3–7	21	6	4–8	0.055
Implantation-weaning test 2 delay (days)	3	6	5–16	5	5	3–7.5	2	5	5–5	0.666
Implantation-weaning test 3 delay (days)	3	6	5–8	2	5	5–5	2	8	7–9	0.103
ECMO weaning-death delay for death < 1 year (days)	20	10	5–13	4	9	3.5–15	16	9.5	4.5–13	0.999
ECMO weaning-hospital discharge delay (days)	30	20	11–34	29	19	11–33	1	55	55–55	NA
Weaning—ICU discharge delay (days)	32	7	5–14	29	19	11–33	1	55	55–55	NA
Duration of ICU stay at weaning (days)	54	6	4–8	36	5	4–7	17	7	5–9	0.223
Duration of inotropic support at weaning (days)	54	3	1–5	36	3	1–5	17	2	1–7	0.624
Duration of vasopressor support at weaning (days)	53	2	1–4	35	2	1–4	17	3	2–7	0.072
Duration of MV support at weaning (days)	54	6	4–8	36	5	4–7	17	7	5–9	0.167
RRT (%)	57	11 (19%)		36	3 (8%)	21		8 (38%)		**0.012**
Levosimendan (%)	57	5 (9%)		36	3 (8%)	21		2 (10%)		0.999

Significant "p" values are highlighted.

*ECMO* extracorporeal membrane oxygenation, *ICU* intensive care unit, *MV* mechanical ventilator, *RRT* renal replacement therapy.

At the weaning test start, all patient presented “weaning criteria”, i.e. blood pressure pulsatility, MBP above 65 mmHg, low catecholamine doses, adequate oxygenation, and ECMO flow below 2 l/min as requested (Table 2).

**Table 2:** Comparison of pre-test and post-test clinical, biological and echocardiographic data according to the success or failure of VA ECMO weaning at 30-day.

Weaning test 1	0verall n = 57			Weaning success n = 36 (63.2%)			Weaning failure n = 31 (36.8%)			P^1^	P^2^ Tous	P^3^ SC	P^4^ EC
	n	Median	25–75 P	n	Median	25–75 P	n	Median	25–75 P	Compair 1–1 et 2–2	Compair 1–2	Compair 1–2	Compair 1–2
**Clinical parameters**													
SBP 1 (mmHg)	57	115	104–129	36	119	111–134	21	114	97–127	0.069	0.977	0.416	0.312
SBP 2 (mmHg)	57	117	107–134	36	128	114–136	21	110	101–119	0.002*			
DBP 1 (mmHg)	57	68	60–76	36	70	62–78	21	67	58–73	0.185	0.006*	0.02*	0.134
SBP 2 (mmHg)	57	66	60–72	36	68	61–73	21	60	55–70	0.089			
MBP 1 (mmHg)	57	84	75–89	36	85	79–91	21	77	73–85	0.020*	0.035*	0.164	0.143
MBP 2 (mmHg)	57	82	74–89	36	85	78–90	21	74	70–83	0.003*			
Pulse pressure 1 (mmHg)	57	50	39–59	36	51	40–59	21	47	36–59	0.313	0.002*	< 0.001*	0.768
Pulse pressure 2 (mmHg)	57	52	43–64	36	57	47–65	21	48	42–55	0.043*			
HR 1 (/min)	57	94	85–103	36	94	85–103	21	94	85–102	0.928	0.009*	0.022*	0.349
HR 2 (/min)	57	98	88–105	36	98	88–106	21	98	86–106	0.798			
ECMO flow 1 (L/min)	57	2.8	2.5–3.3	36	2.8	2.4–3.2	21	2.9	2.5–3.5	0.337	< 0.001*	< 0.001*	0.001*
ECMO flow2 (L/min)	57	1.6	1.5–1.8	36	1.6	1.5–1.6	21	1.6	1.5–1.8	0.244			
Dobutamine 1 (γ/kg/min)	26	5	3.8–5	15	5	4.1–5	11	5	3.1–6.9	0.9778	0.6639	NA	NA
Dobutamine 2 (γ/kg/min)	25	5	3.6–6.4	15	5	4.1–5.8	10	5	2.5–7.5	0.8842			
Noradrenaline 1 (mg/h)	13	0.8	0.5–1.05	8	0.85	0.6–1	5	0.5	0.5–2.1	0.8828	0.3125	NA	NA
Noradrenaline 2 (mg/h)	13	0.8	0.5–1.2	8	0.8	0.6–1.1	5	0.5	0.4–2.1	0.6074			
**Biological parameters**													
PT (%)	50	73	61–83	33	75	64–84	17	66	59–81	0.448	NA	NA	NA
Hb (%)	57	9	8.1–9.6	36	9.2	8.4–9.7	21	8.2	8–9.7	0.103	NA	NA	NA
Pl (G/l)	57	89	67–113	36	89	81–115	21	82	55–111	0.18	NA	NA	NA
lactate 1 (mmol/l)	45	1.1	0.8–1.6	29	1	0.8–1.5	16	1.2	0.9–1.7	0.318	0.026*	0.036*	0.459
lactate 2 (mmol/l)	46	1	0.7–1.3	31	0.9	0.7–1.3	15	1	0.7–1.4	0.697			
RR 1 (/min)	50	18	16–22	30	18	16–22	20	19	17–22	0.913	0.065	< 0.001*	0.733
RR 2 (/min)	51	19	16–24	31	20	16–24	20	19	17–23	0.885			
pH 1	57	7.45	7.4–7.49	36	7.45	7.4–7.48	21	7.45	7.4–7.5	0.545	0.066	0.212	0.182
pH 2	55	7.47	7.4–7.52	35	7.47	7.41–7.51	20	7.49	7.4–7.5	0.233			
HCO_3_^−^ 1 (mmol/l)	57	24.7	22.2–26.6	36	24.7	22–26.6	21	24.2	23–26.4	0.856	0.577	0.713	0.284
HCO_3_^−^ 2 (mmol/l)	56	24.4	22–26.5	36	24.7	21.8–26.5	20	23.9	23–26.8	0.732			
paO_2_ 1 (mmHg)	57	104	84–132	36	104	80–142	21	105	88–122	0.856	0.014*	0.026*	0.177
PaO_2_ 2 (mmHg)	55	84	75–117	35	83	75–117	20	84	74–116	0.834			
pCO_2_ 1 (mmHg)	57	36	31–40	36	37	32–40	21	35	31–40	0.568	0.076	0.112	0.374
pCO_2_ 2 (mmHg)	55	34	30–38	35	34	30–39	20	34	30–37	0.568			
SVO_2_ 1 (%)	49	71	59–78	32	72	58–80	17	71	58–73	0.248	0.014*	0.202	0.027*
SVO_2_ 2 (%)	40	66	58–72	24	68	60–72	16	62	55–71	0.194			
**Echographic parameters**													
LVEF 1 (%)	45	28	20–40	27	35	25–44	18	24	20–31	0.009*	0.002*	< 0.001*	0.244
LVEF 2 (%)	45	30	25–45	26	38	27–55	19	25	21–34	0.002*			
IVC 1 (mm)	44	21	18–24	28	22	19–25	16	20	17–22	0.13	0.845	0.756	0.52
IVC 2 (mm)	43	21	18–25	28	23	19–25	15	20	18–23	0.609			
Ea 1 (cm/s)	54	8.6	5.3–11	34	9	6–11	20	7.2	5–10	0.262	< 0.001*	0.014*	0.002*
Ea 2 (cm/s)	53	9.8	7–12	33	9.8	8–13	20	9.5	7–11	0.55			
Aortic VTI 1 (cm)	53	12	10.5–14	32	12	11–16	21	12	10–13	0.199	< 0.001*	0.002*	< 0.001*
Aortic VTI 2 (cm)	52	14	12–17	31	15	12–16	21	14	12–17	0.758			
Pulm VTI 1 (cm)	39	12.5	10–15.5	24	13.6	10–16	15	11	9–14	0.115	0.136	0.265	0.454
Pulm VTI 2 (cm)	40	13	11–16	23	14	11–16	17	12	9–16	0.118			
A 1 (cm/s)	37	57	41–67	21	57	41–65	16	51	39–86	0.963	0.31	0.611	0.339
A 2 (cm/s)	42	54	35–70	24	56	36–70	18	50	35–70	0.929			
E 1 (cm/s)	47	75	60–90	28	76	63–93	19	75	60–87	0.803	0.009*	0.131	0.017*
E 2 (cm/s)	51	80	68–95	31	81	65–95	20	78	68–95	0.772			
E1/Ea1	45	8.4	6.6–12	27	7.7	6–12	18	11	7.6–13	0.105	0.023*	0.191	0.062
E2/Ea2	49	8	6–12	30	7.5	6–11	19	8.5	6.5–12	0.587			
SRV 1 (cm/s)	54	11	8–13	34	11	8–14	20	9	8–11	0.228	0.004*	0.022*	0.094
SRV 2 (cm/s)	53	12	9–14	35	13	10–15	18	12	9–14	0.278			
SLV 1 (cm/s)	54	6.6	5–9	34	7	5–10	20	6	5–9	0.564	0.003*	0.011*	0.098
SLV 2 (cm/s)	52	7.2	6–11	32	9	6–11	20	7	6–10	0.282			
TAPSE 1 (mm)	54	17.8	13–20	34	18	13–20	20	16	15–20	0.9	0.007*	0.039*	0.064
TAPSE 2 (mm)	53	19	16–22	33	19	15–22	20	19	17–22	0.956			

P1: comparison of the parameter of each group: SWG vs FWG (Mann–Whitney).

P2 P3 P4: evolution of the parameter at the 2 times (pre and post-test) of the test in each of the groups (Wilcoxon).

Significant “p” values are highlighted.

*A* mitral annulus peak systolic velocity, *Aortic VTI* sub-aortic time-velocity integral, *DBP* diastolic blood pressure, *E* transmitral early peak diastolic velocity, *Ea* spectral tissue Doppler lateral mitral annulus peak systolic early annular velocity, *ECMO* extracorporeal membrane oxygenation, *Hb* hemoglobin, *HCO*_*3*_^−^ bicarbonate, *HR* heart rate, *IVC* inferior vena cava, *LVEF* left ventricular ejection fraction, *MBP* mean blood pressure, *PaO*_*2*_ arterial partial pressure of oxygen, *PaCO*_*2*_ arterial partial pressure of carbon monoxide, *Pl* platelets, *PT* prothrombine time, *Pulmonary VTI* sub-pulmonary time-velocity integral, *RR* respiratory rate, *SBP* systolic blood pressure, *S*′* LV* S wave at the mitral annulus, *S*′* RV* S wave at the tricuspid annulus, *SVO*_*2*_ venous oxygen saturation, *TAPSE* tricuspid annular systolic excursion in TM mode.

### Predictors of weaning test failure

#### Static analysis

Pre-test clinical variables were comparable between groups, except for the MBP, which was higher in the SWG (85 vs 77 mmHg; p = 0.02). At baseline, numbers of patients on noradrenaline or dobutamine were comparable between groups (5/21 (24%) vs 8/36 (22%); p = 0.89 and 10/21 (48%) vs 15/36 (42%), p = 0.67, respectively for FWG and SWG). Post-test MBP, SBP and pulse pressure were significantly increased in the SWG (85 vs 74 mmHg (p = 0.003); 128 vs 110 mmHg (p = 0.002) and 57 vs 48 mmHg (p = 0.043), respectively). Biological data for the 2 groups were comparable at each time of the test. Regarding echocardiographic data, only LVEF was higher in the SWG both in pre (35 vs 24%, p = 0.009) and post- test (38 vs 25%; p = 0.002) (Table 2).

#### Dynamic analysis

**Figure 2:**
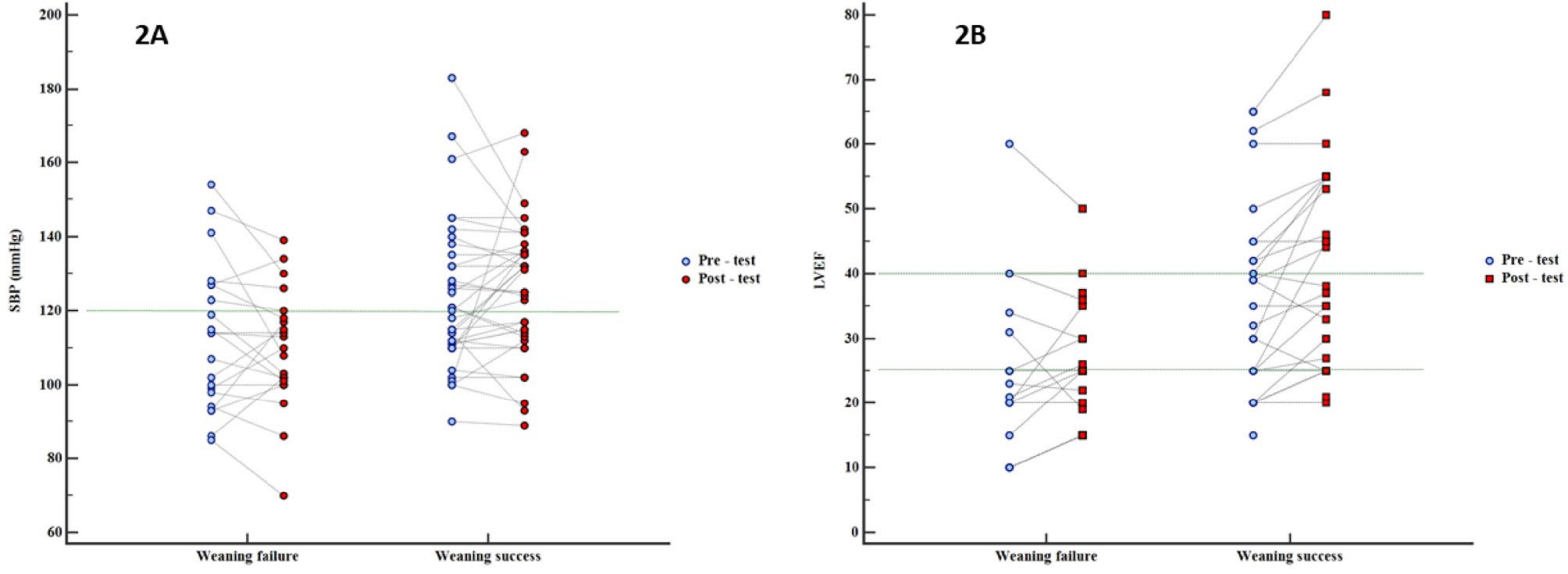
Evolution of systolic blood pressure (**A**) and LVEF (**B**) during the weaning test depending on the success or failure of weaning. Pre-test parameters are represented as blue point and post-test results in red. p value for comparisons between pre and post-test parameters and between successful weaning and failure weaning test groups are presented in Table 2. *DBP* diastolic blood pressure, *LVEF* left ventricular ejection fraction.

During the weaning test, Fig. 2A and Table 2 show a significant decrease in blood pressure in the FWG (p = 0.006 for DBP and p = 0.035 for MBP) whereas we observed a significant increase in pulse pressure in the SWG (p = 0.002). Lactates’ levels significantly decreased in the SWG whereas ScVO_2_ decreased significantly in the FWG [respectively from 1 to 0.9 mmol/l (p = 0.036) and from 71 to 62% (p = 0.027)].

Echocardiographic data showed a more significant increase of LVEF in the SWG (from 35 to 38% vs 24 to 25% respectively, p = 0.002) (Fig. 2B). Similarly, the sub-aortic velocity time integral, the S wave at the lateral mitral annulus, E wave, and Ea wave increase were significantly greater in the SWG (p = 0.003, p = 0.009 and p < 0.001 respectively). Right ventricular function defined by the S wave at the tricuspid annulus and the tricuspid annular systolic excursion (TAPSE) improved significantly in the SWG [respectively from 11 to 13 cm/s (p = 0.004) and from 18 to 19 mm (p = 0.007)].

As showed in the Supplementary Table 2, the ROC curves defined several pre-test (MBP ≤ 83 mmHg; LVEF ≤ 25% and total time on vasopressors > 4 days) and post-test (SBP ≤ 120 mmHg, pulse pressure ≤ 54 mmHg and LVEF ≤ 40%) variables of interest.

#### Multivariate analysis

Multivariate analysis revealed three predictors of weaning failure: a post-test SBP ≤ 120 mmHg, a delay in ECMO weaning > 7 days and a history of ischemic heart disease (Table 3). LVEF can also be taken into account with a pre-test LVEF ≤ 25% and/or a post-test LVEF ≤ 40% (p = 0.052). This value does not reach significance but seems to discriminate in patient outcome after weaning. This logistic regression model classifies 81.6% of the cases correctly.

**Table 3:** Multivariate analysis of predictors of weaning failure at 30-day after first weaning test performed.

Weaning failure	OR	IC 95%	p
Post-test SBP ≤ 120 mmHg	33	3–385	**0.005**
Pre-test LVEF ≤ 25% and/or post-test LVEF ≤ 40%	11	0.98–115	0.052
Implantation-weaning test delay > 7 days	24	2–269	**0.011**
Pre-existing ischemic cardiopathy	9.6	1.1–83	**0.04**
AUC	0.93 [0.82–0.98]		
Hosmer Lemeshow test	0.52		
Percentage of correctly classified cases	81.6%		

The results of the multivariate analysis presented here were obtained only with the pre-test data and those obtained during the first weaning test.

Significant “p” values are highlighted.

*AUC* area under the curve, *LVEF* left ventricular ejection fraction, *SBP* systolic blood pressure.

### Decision-making model

**Figure 3:**
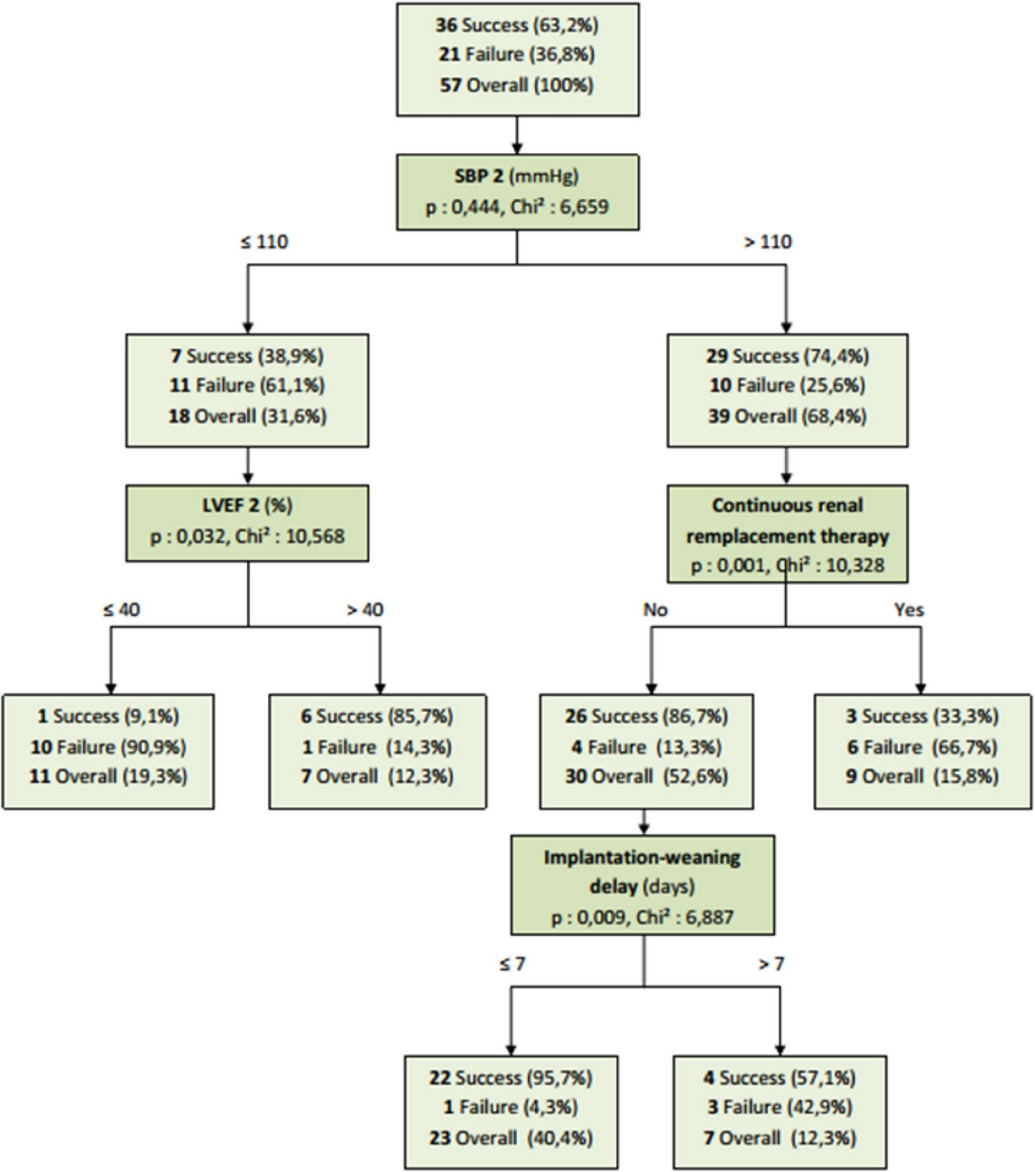
Decision tree (classification tree diagrams). For example, patients who had a post-test SBP of less than 110 mmHg and a post-test LVEF less than or equal to 40% had a 90.9% chance of weaning failure on D30, whereas those with a post-test SBP of more than 110 mmHg who were not dialyzed with a time to weaning of less than 7 days, had a 95.7% chance of weaning success on D30. *LVEF* left ventricular ejection fraction, *SBP* systolic blood pressure.

The goal of this decision tree method was to identify the most important risk factors from a pool of potential risk factors selected in the bivariate and multivariate analysis. The decision tree model generated from the dataset is shown in Fig. 3 which illustrates the importance of evaluating LVEF and hemodynamic conditions (SBP) in weaning decision. We used the CHAID approach which allows classification of 84.2% of the patients in the correct category (validation after analysis on a training sample with a (pp) at 84.6% and then on a test sample with a (pp) at 74.2%).

## Discussion

In our prospective registry of patients implanted with VA ECMO for whom a systematic weaning test was performed when the clinician considered it feasible, several points are highlighted: (a) 54% of the patients were successfully weaned after the first test (i.e., alive with no need for a new VA ECMO or heart transplantation or LVAD at 30 days); (b) in case of failure of the first test, redo the test is pivotal, since 3 on 7 could be weaned after the 2nd and 3 on 4 after the 3rd test; (c) the existence of preexisting ischemic heart disease (OR 9.6 [1, 1–83]), pre-test LVEF ≤ 25% or post-test LVEF ≤ 40% (OR 11 [0.98–115]), post-test SBP ≤ 120 mmHg (OR 33 [3–385]), or an implantation-weaning test period > 7 days (OR 24 [2–269]) should raise concerns about weaning failure.

In accordance with the literature^8,9,24^, the weaning test and inclusion in the study called for several prerequisites (hemodynamic and respiratory stability) introducing part of subjectivity tending to select a population with higher probability of weaning success. As in several previous reports, the decision to wean the ECMO was left to the discretion of the clinician who was blinded to the weaning test in our study^9,15,25^, whereas others weaned all patients using the prerequisites mentioned above^8,13^.

This explains that several patients underwent multiple weaning tests before being weaned but 86.6% (n = 31) of the successful weaning occurred after the first test, whereas only 8% (n = 3) after the 2nd and 6% (n = 2) after the 3rd, underlying the experience of the clinician in charge of the patient. Moreover, a total of 19 patients (90% of the patients for whom weaning failed) did not have multiple tests because the patient died or was then referred to transplant or LVAD before another test could be performed. This is especially important for VA ECMO patients in France since January 2018 and the new allocation graft score used. At ECMO implantation, patients have the maximum chance to access to a heart graft but after day 12, chance decreases and patients are definitively removed from the transplant list on day 16. This new legislation may change practices and influence the decision of whether or not to attempt to wean the patient and especially its timing. That is why it is important to have well-defined parameters helping us to quickly identify which patient can be weaned and for which other it is futile to attempt weaning.

Our weaning success rate (63%) is consistent with the literature (between 35 and 70%), even though these rates are variable due to different populations, prerequisites and definitions of success used^5,8–10,15,20,23,24^. In older studies, the success rate was lower (≈ 40%)^8,23^, whereas recent studies with selected patients suitable for weaning had similar results (60–70%)^10,24^. This selection of patients considered as suitable for weaning by the clinician, explains the low number of non-weaned patients after the first test (n = 10; 18%). In addition, our definition of successful weaning as a weaned patient with no need for further VA-ECMO, transplantation or LVAD at 30 days has strong clinical significance when the decision is made at bedside, unlike the majority of criteria used to date (survival without support at 48 h, 30 days, or at discharge).

The predictive criteria of weaning failure that we reported are clinically relevant since even before the weaning test, some simple clinical markers can already indicate risk of failure, such as a history of ischemic heart disease, a low MBP under ECMO (< 83 mmHg) or a minimum LVEF < 25%. Similarly, the course of the ICU stay before the test, indicates the probability of weaning failure in case of RRT use, prolonged period of mechanical ventilation, time on vasopressor > 4 days and time between ECMO implantation and the weaning test > 7 days. The weaning test provides additional elements such as post-test SBP < 120 mmHg which was the main predictive factor of weaning failure (OR = 33 [3–385]). Finally, dynamic analysis demonstrated that blood pressure (MBP, DBP) decreased more significantly when weaning fails and conversely, pulse pressure increased less or not at all.

The TTE played an important role based on simple and easily accessible criteria by non-expert intensivist in these complex ICU patients. Pre-test LVEF < 25% and/or post-test LVEF < 40%were strong markers of weaning failure (OR 11 [0.98–115]) and dynamic analysis showed that an increase in LVEF during testing was predictive of success. A previous single-center prospective study of 51 patients found others TTE markers associated with VA ECMO weaning success (LVEF > 20–25%, sub-aortic VTI > 10 cm and a Lat LV S′ > 6 cm/s) but these thresholds were set arbitrarily^8^. In our series, sub-aortic VTI and Lat LV S′ did not appear to be discriminating, probably because of a lack of power, particularly related to missing data. But our dynamic analysis showed increase in the sub-aortic VTI and Lat LV S′ as well as decrease in the E/Ea ratio during the test indicating a systolic as well as a diastolic adaptation of the left ventricle during re-loading in the SWG.

The right ventricle should also be studied since the decrease in ECMO flow is responsible for an increase in RV preload and the re-loading of a failing LV may also be responsible for an increase in RV afterload. Some authors showed an association of RV ejection fraction (> 24.6%) assessed by 3D ultrasound with successful weaning and decreased 30-day mortality^15^. Others showed interest for parameters that include the combined analysis of non-invasive parameters of RV function and pressures, as well as of pulmonary circulation, such as the tricuspid annulus S wave/right ventricular systolic pressure (RVSP) ratio (> 0.33), TAPSE/RVSP and the free wall longitudinal strain/RVSP^24^. These parameters, seem to show good diagnostic performances but are also limited by the way they are performed, requiring an expert echocardiogram with expert sonographer as well as a satisfactory ultrasound window limiting the clinical applicability in intensive care patients. We did not find statistically significant difference in the simple parameters of right ventricular function assessment (TAPSE and RV S′), neither pre- nor post-test between groups, although these parameters improved significantly during the test in the SWG. A lack of power and missing data prevented a definitive conclusion on these parameters. Finally, RV/LV interdependence and RV dilatation during the weaning test seem to be an even simpler parameter to assist in the decision of whether or not our patients may be candidates for weaning, but was not studied here and will be confirmed in larger studies^14^.

In order to facilitate the clinical approach, we modeled a segmentation tree, which allowed the prediction of weaning failure by integrating pre- and post-test parameters with a prediction rate of 84.6% based on simple parameters easily available at the patient bedside (post-test LVEF, current RRT, ECMO placement-weaning period and post-test SBP).

In the future, these different static and dynamic parameters could be included in weaning protocols in the same way as hemodynamic and respiratory stability, which are prerequisites for the test. It appears necessary to carry out larger multicenter studies with a repetition of systematic weaning tests in case of failure in order to establish robust predictive criteria for VA ECMO weaning.

### Limitations

First, this is a monocentric study conducted in an expert tertiary reference center, limiting its external extrapolation, especially regarding the wide heterogeneity of protocols of VA ECMO management among teams.

Secondly, this is a heterogeneous population in terms of ECMO indications, as often observed in the literature. We did not include post-cardiotomy patients in our medical intensive care unit, limiting any extrapolation to this very specific population.

Third, we decided to consider only deaths from cardiovascular causes (not cerebral anoxia secondary to CA) in order to focus on circulatory support and its weaning criteria, explaining why 4 patients in the SWG were dead before 30-day and not considered in the FWG (Table 1).

Fourth, patients were included when they were considered by the clinician in charge to be candidates for weaning. Therefore, although the eligibility criteria were predefined [hemodynamic and respiratory stability in particular (see “Materials and methods”)], a certain level of subjectivity persists, leading to a possible selection bias.

In addition, although it remains one of the largest series published to date, our low number of patients (n = 57) and some missing data limit the statistical analyses and therefore our results, particularly concerning the additional weaning tests (tests 2 and 3).

Finally, we defined the weaning test as a decrease in ECMO flow below 2 l/min with a median flow at the end of the test of 1.6 l/min which is consistent with previous reports (1–1.5 l/min)^14,21^ but higher than more recent studies (0.5–1 l/min)^22,24^. It is possible that a greater flow rate reduction would have provided different, more sensitive or specific results. However, exposing the patient to a greater reduction in flow (or even to clamp tests (“trial Off ECMO”) as done by some teams) exposes the patient to a thrombotic risk or even hemorrhage in case of an associated bolus of anticoagulant, which is difficult to evaluate.

## Conclusion

VA ECMO weaning failed in less than 40% of patients considered suitable for weaning. In case of failure, further weaning test should be considered. Pre-existing ischaemic heart disease, ECMO duration for more than 7 days, pre-test LVEF ≤ 25%, post-test LVEF ≤ 40% and/or SBP < 120 mmHg following an optimized weaning test should trigger further review. The use of these criteria may help clinical decision-making during the weaning process although we acknowledge the limitations of a single-centre study. A multi-centre study may be required to ascertain the validity of the proposed criteria.

## Supplementary Information

Supplementary Tables.

## Data Availability

The datasets used and/or analyzed during the current study are available from the corresponding author upon reasonable request.
