# Supplementary material for: Predictors of weaning failure in case of VA ECMO implantation
Source: Sci Rep. 2022 Aug 16;12:13842. doi: 10.1038/s41598-022-18105-y (PMC9381562; doi:10.1038/s41598-022-18105-y)
Supplement: Supplementary file 1 — Supplementary Tables. [file 41598_2022_18105_MOESM1_ESM.docx]

**Supplemental data: “Predictors of weaning failure in case of VA ECMO implantation” Cusanno.A et al**

**Supplemental material and methods**

Clinical and echocardiographic parameters recorded before and after the weaning test

Clinical (heart rate, systolic blood pressure (SBP), diastolic blood pressure (DBP), mean blood pressure (MBP), differential pressure (SBP - DBP), blood pressure pulsatility, RASS score) and therapies implemented (dobutamine, noradrenaline, adrenaline, levosimendan, sedative, IABP, mechanical ventilation, and renal replacement therapy (RRT)), VA-ECMO parameters (flow, gas flow and FiO2) and biological data (arterial blood gas and lactates, mixed venous oxygen saturation (SvO2), creatinine, PT, platelet and blood cell count) were also collected at each time.

Visual LVEF (%), mitral annular velocity (lat LV S’), sub-aortic velocity time integral (VTI), early-diastolic mitral wave velocity by pulsed Doppler (E), end-diastolic mitral wave velocity by pulsed Doppler (A), early-diastolic mitral annular velocity by tissue Doppler (Ea), sub-pulmonary velocity time integral (VTI), tricuspid annular velocity par tissue Doppler (RV S’), tricuspid annular plane systolic excursion (TAPSE), and inferior vena cava diameter were recorded.

**Supplemental data results**

**Supplemental table 1: Comparison of baseline and management characteristics according to the VA ECMO weaning success or failure at 30-day**

|  | **Overall population**  **n=57** | | | **Successful Weaning group**  **n=36** | | **Failure weaning group**  **n=21** | | **P** |
| --- | --- | --- | --- | --- | --- | --- | --- | --- |
|  | N |  |  | N | | N | |  |
| **Baseline characteristics, median (IQR 25-75)** |  |  | |  |  |  |  |  |
| **Age (yo)** | 57 | 50 (37.7 to 58) | | 36 | 46 (38 to 57) | 21 | 57 (42 to 59) | 0.143 |
| **BMI (kg/m^2^)** | 57 | 26 (23 to 29) | | 36 | 26 (23 to 29) | 21 | 25 (23 to 30) | 0.83 |
| **LVEF-min (%)** | 57 | 10 (5 to 11) | | 36 | 10 (5 to 15) | 21 | 10 (5 to 10) | 0.687 |
|  |  |  | |  |  |  |  |  |
| **Previous cardiac arrest n (%)** | 57 | 16 (28.1%) | | 36 | 8 (22.2 %) | 21 | 8 (38.1%) | 0.202 |
| **VA ECMO indication n (%)** |  |  | |  |  |  |  |  |
| **- Post myocardial infarction RCS** | 57 | 20 (35.1%) | | 36 | 14 (38.9%) | 21 | 6 (28.6%) | 0.435 |
| **- Advanced heart failure** | 57 | 5 (8.9%) | | 36 | 2 (5.6%) | 21 | 3 (15%) | 0.336 |
| **- Drug intoxication** | 57 | 4 (7%) | | 36 | 4 (11%) | 21 | 0 (0%) | 0.285 |
| **- Refractory cardiac arrest** | 57 | 16 (28.1%) | | 36 | 8 (22.2%) | 21 | 8 (38.1%) | 0.202 |
| **- Myocarditis or other** | 57 | 9 (15.8%) | | 36 | 7 (19.4%) | 21 | 2 (9.5%) | 0.326 |
| **Known cardiomyopathy** |  |  | |  |  |  |  |  |
| **-** **None** | 57 | 37 (64.9%) | | 36 | 26 (72.2%) | 21 | 11 (52.4%) | 0.133 |
| **- Ischemic cardiomyopathy** | 57 | 13 (22.8%) | | 36 | 5 (13.9%) | 21 | 8 (38.1% | **0.037** |
| **- Dilated cardiomyopathy** | 57 | 7 (12.3%) | | 36 | 2 (5.6%) | 21 | 5 (23.8%) | 0.088 |
| **- Valvular cardiomyopathy** | 57 | 5 (8.8%) | | 36 | 2 (5.6%) | 21 | 3 (14.3%) | 0.346 |
| **- Rythmic cardiomyopathy** | 57 | 5 (8.8%) | | 36 | 2 (5.6%) | 21 | 3 (14.3%) | 0.346 |
|  |  |  | |  |  |  |  |  |
| **Peripheral artery disease** | 57 | 14 (24.6%) | | 36 | 11 (30.6%) | 21 | 3 (14.3%) | 0.214 |
| **Diabetes** | 57 | 8 (14%) | | 36 | 4 (11.1%) | 21 | 4 (19%) | 0.449 |
| **Dyslipidemia** | 57 | 8 (14%) | | 36 | 3 (8.3%) | 21 | 5 (23.8%) | 0.13 |
| **Obesity** | 57 | 9 (16.1%) | | 36 | 5 (14.3%) | 21 | 4 (19%) | 0.715 |
| **Current smoking** | 57 | 24 (42 %) | | 36 | 17 (47.2%) | 21 | 7 (33.3%) | 0.031 |
| **Biological parameters at ECMO implantation:**  **median (IQR 25-75)** |  |  | |  |  |  |  |  |
| **Creatinine (mmol/l)** | 56 | 120.5 (97 to 168) | | 35 | 114 (93 to 166) | 21 | 124 (100 to 177) | 0.379 |
| **Hemoglobin (g/dl)** | 56 | 11.7 (10.2 to 14) | | 35 | 11.8 (10 to 14.2) | 21 | 11.6 (10.3 to 13.2) | 0.612 |
| **Lactate (mmol/l)** | 53 | 6.7 (3.8 to 10) | | 34 | 6.35 (4.5 to 10.7) | 19 | 6.7 (3.5 to 8.5) | 0.67 |
| **PaCO2 (mmHg)** | 56 | 35.8 (30.1 to 42.8) | | 35 | 36 (29 to 45) | 21 | 36 (31 to 41)) | 0.729 |
| **PaO2 (mmHg)** | 56 | 225 (103.8 to 335) | | 35 | 208 (103 to 299) | 21 | 234 (123 to 459) | 0.26 |
| **pH** | 56 | 7.3 (7.16 to 7.42) | | 35 | 7.29 (7.1 to 7.4) | 21 | 7.3 (7.2 to 7.4) | 0.379 |
| **HCO3- (mmol/l)** | 56 | 17.8 (13 to 20) | | 35 | 17 (13 to 20) | 21 | 18 (14 to 20) | 0.493 |
| **Platelets (G/l)** | 56 | 190 (130 to 247) | | 35 | 183 (123 to 241) | 21 | 191 (138 to 249) | 0.542 |
| **Prothrombin time (%)** | 53 | 55 (44 to 64) | | 33 | 57 (44 to 64) | 20 | 55 (49 to 62) | 0.833 |
| **Parameters concerning ICU stay: median (I**QR **25-75)** |  |  | |  |  |  |  |  |
| **Total stay in ICU (days)** | 57 | 15 (12 to 25) | | 36 | 14 (12 to 24) | 21 | 17 (12 to 26) | 0.345 |
| **Duration of inotropic support (days)** | 57 | 4 (2 to 7) | | 36 | 4 (2 to 6) | 21 | 5 (2 to 14) | 0.173 |
| **Duration of vasopressor support (days)** | 56 | 4 (2 to 7) | | 35 | 2 (1 to 4) | 21 | 6 (4 to 9) | **0.006** |
| **Duration of MV support (days)** | 57 | 11 (8 to 17) | | 36 | 10 (7 to 16) | 21 | 15 (10 to 18) | **0.033** |

Significant "p" values are highlighted.

BMI, body mass index; ECMO, Extracorporeal membrane oxygenation; ICU, intensive care unit; IQR, interquantile range; Pa02, Arterial partial pressure of oxygen; PaCO2, arterial partial pressure of carbon monoxide; RCS, Refractory cardiogenic shock; MV, mechanical ventilator

**Supplementary table 2: AUC curves, sensitivity, specificity, positive and negative predictive values of the variables of interest on bivariate analysis to predict the weaning failure at 30-day**

| **Variable** | **AUC** | **CI 95%** | **P** (Area=0.5) | **Criterion** | **Sensitivity** | **Specificity** | **PPV** | **NPV** |
| --- | --- | --- | --- | --- | --- | --- | --- | --- |
| **Implantation – weaning test 1 delay** | 0.65 | 0.51 to 077 | 0.05 | >7 | 47.6 | 86.1 | 66.7 | 73.8 |
| **MBP 1-SEV1** | 0.69 | 0.55 to 0.80 | **0.013** | ≤83 | 71.4 | 63.9 | 53.6 | 79.3 |
| **PAS 2-SEV1** | 0.74 | 0.61 to 0.85 | **< 0.002** | ≤120 | 80.9 | 61.1 | 54.8 | 84.6 |
| **Pulse pressure 2-SEV1** | 0.66 | 0.52 to 0.78 | **0.03** | ≤54 | 76.2 | 61.1 | 53.3 | 81.5 |
| **Total duration of vasopressor support** | 0.72 | 0.58 to 0.83 | **0.007** | >4 | 71 | 80 | 68 | 82.4 |
| **LVEF 1-SEV1** | 0.73 | 0.58 to 0.85 | **0.002** | ≤25 | 72.2 | 66.7 | 59.1 | 78.3 |
| **LVEF 2-SEV1** | 0.77 | 0.62 to 0.89 | **< 0.001** | ≤40 | 94.7 | 46.2 | 56.2 | 92.3 |

Significant "p" values are highlighted.

DBP, Diastolic blood pressure ; LVEF, Left ventricular ejection fraction ; MBP, Mean blood pressure ; NPV, Negative predictive value ; PPV, Positive predictive value ; SBP, Systolic blood pressure ; SEV1, Weaning test 1
